# High-resolution model experiments unveil the crucial role of small-scale air–sea interactions in large-scale oceanic water mass formation

**DOI:** 10.1093/nsr/nwad184

**Published:** 2023-06-28

**Authors:** Shoshiro Minobe

**Affiliations:** Department of Earth and Planetary Sciences, Faculty of Science, Hokkaido University, Japan

Subtropical mode water (STMW) plays a crucial role in the Earth’s climate, including the oceans’ absorption of anthropogenic CO_2_ and heat produced by global warming, thus slowing the progression of global warming. Therefore, accurate prediction of STMW is important for future climate change projections. However, there has been a problem with the climate models that have been used for climate projections in that they have not been able to adequately reproduce STMW. The amount of STMW in the climate models is too small.

Gan *et al.* (2023) [1] demonstrated the importance of frontal-scale atmosphere–ocean interactions, which are active in high-resolution models with eddy-rich oceans and less so in low-resolution models with eddy-free oceans, in the formation of STMWs by performing highly sophisticated coupled atmosphere–ocean model experiments. They evaluated the effect of frontal-scale oceanic variations as perceived by the atmosphere by performing two sets of experiments—one with an eddy-rich coupled atmosphere–ocean model (sometimes referred to as eddy-resolving), and another in which frontal-scale air–sea interactions were removed by applying a spatial low-pass filter to the sea surface temperature (SST) sensed by the atmosphere. The results were striking; in the experiment where the atmosphere did not sense the frontal-scale oceanic features, the production of STMW was dramatically reduced by half.

They also explained why frontal-scale air–sea interactions are important. In high-resolution models, latent heat release is enhanced over frontal-scale warm SSTs by two effects: increased air–sea moisture contrast and increased wind speed. This enhanced latent heat release from the ocean contributes to vigorous vertical mixing of seawater, creating a larger amount of STMW compared to the case where the frontal-scale air–sea interaction is suppressed. They further analyzed the outputs of the High-Resolution Model Intercomparison Project and the Ocean Model Intercomparison Project phase 2, and obtained consistent results.

These results demonstrate that small, frontal-scale air–sea interactions have an impact on water mass formation in the North Atlantic, which occurs on a much larger scale. The findings add new significance to the importance of frontal-scale air–sea interactions, including aspects that have been overlooked even in recent reviews [2–4].

The present paper has important implications for our understanding of climate change, because the results of Gan *et al.* (2023) imply that the contribution of STMW to climate change has been underestimated. Since STMW absorbs CO_2_ and heat, if these effects are underestimated, the ocean would be expected to absorb more CO_2_ and heat than is currently thought. On the other hand, if the absorption of CO_2_ is stronger, it is likely that ocean acidification and ocean deoxygenation will become more severe. Furthermore, if the ocean absorbs more heat, marine heatwaves are likely to occur more frequently and the rise of sea-level may be greater. Consequently, if the underestimation of STMW production in climate models is corrected, previously overlooked adverse effects may become apparent.

Therefore, the results of Gan *et al.* (2023) clearly indicate the need for a more accurate representation of frontal-scale air–sea interactions in climate models. To address this issue, as they suggest, it is necessary either to increase the resolution of the models or to apply parameterization to frontal-scale air–sea interactions. Both approaches, however, are likely to require significant effort by the climate science community over a decade or so to achieve sufficient improvements. This effort is essential for understanding and predicting the Earth’s climate, which is shaped and modified by air–sea interactions.

***Conflict of interest statement***. None declared.
